# Preparation and Performance Optimization of an Expansive Backfill Material for Active Roof Contact in End-Wall Mining

**DOI:** 10.3390/ma19163556

**Published:** 2026-08-21

**Authors:** Jinxing Lyu, Zhimeng Song, Bao Song, Yiquan Lin, Wen Ma

**Affiliations:** 1 School of Geology and Mining Engineering, Xinjiang University, Urumqi 830046, China; szm0526@163.com (Z.S.); 107552404741@stu.xju.edu.cn (B.S.); linquan0821@163.com (Y.L.); 107552504754@stu.xju.edu.cn (W.M.); 2 School of Mining Engineering, China University of Mining and Technology, Xuzhou 221116, China

**Keywords:** expansive backfill material, active roof contact, mine overburden, hydrogen peroxide, mechanical properties

## Abstract

Conventional cemented backfill used in narrow end-wall mining entries commonly suffers from shrinkage and insufficient roof contact, reducing its support effectiveness. In this study, a solid-waste-based expansive backfill was prepared from mine overburden, ordinary Portland cement, fly ash and hydrogen peroxide. Orthogonal tests were performed to evaluate the effects of fly ash dosage, aggregate-to-binder ratio and hydrogen peroxide dosage on slurry flowability, expansion ratio and compressive strength. The slurry exhibited a flowability of 17.9–24.7 cm and an expansion ratio of 11.23–46.52%, confirming the gas-generating expansion effect of hydrogen peroxide. The aggregate-to-binder ratio was the dominant factor influencing flowability, expansion and 28 d strength. Multi-index optimization identified an optimal mix of 40% fly ash, an aggregate-to-binder ratio of 4:1 and 10% hydrogen peroxide. The optimized backfill showed age-dependent increases in uniaxial compressive strength (UCS) and elastic modulus, with failure evolving from inclined shear to tensile and tensile–shear composite modes. Its shear behavior followed the Mohr–Coulomb criterion, with cohesion of 1.61 MPa and internal friction angle of 30.13°. SEM observations indicated that aggregate skeleton support, cementitious bonding and gas-generating expansion jointly controlled the material performance.

## 1. Introduction

**Figure 1:**
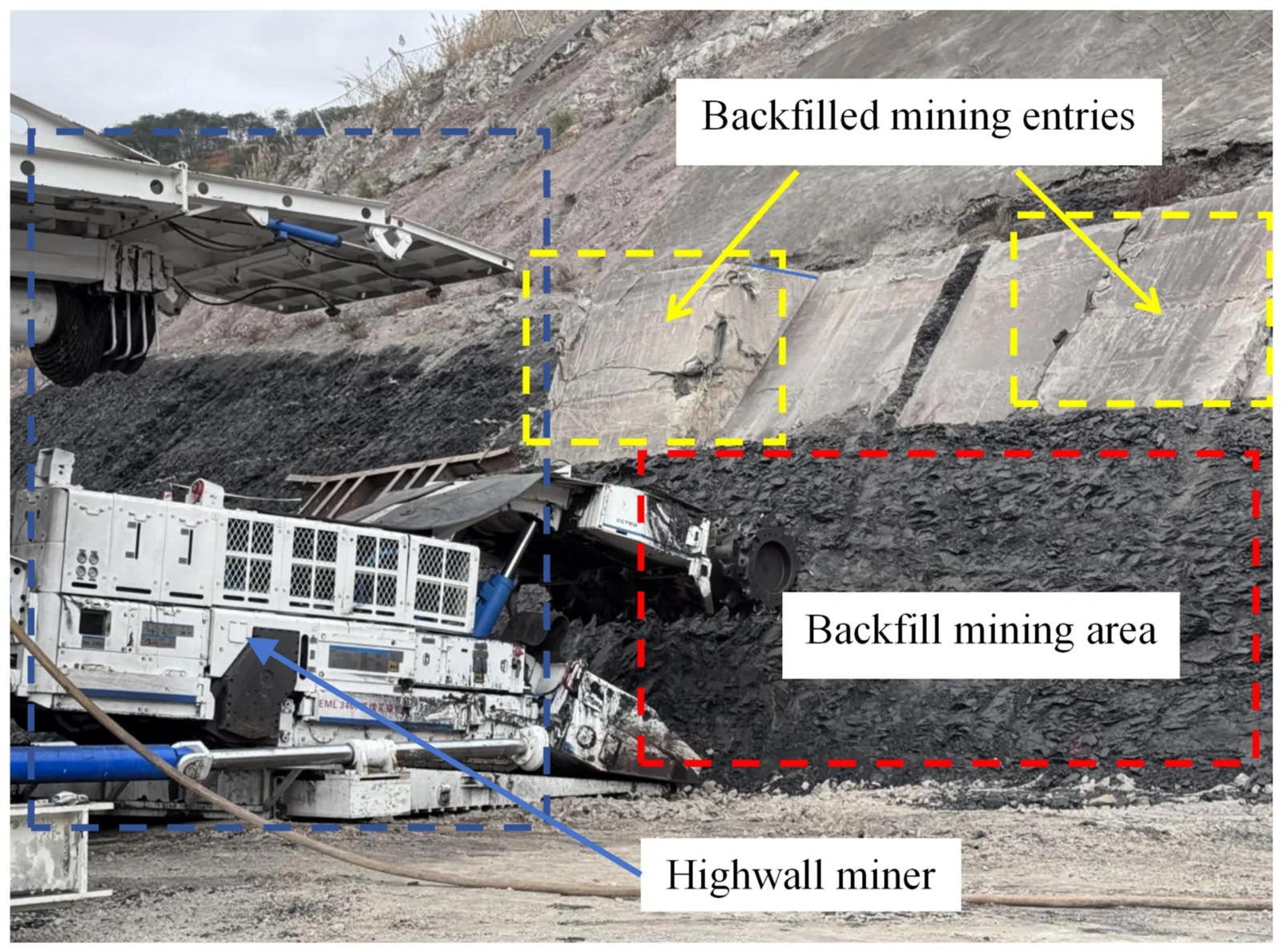
Example of the end-wall coal mining engineering scene.

As open-pit mining gradually advances toward greater depths and more complex boundary conditions, increasing attention has been paid to the recovery of residual coal resources in the end-wall areas of open-pit coal mines [1,2]. In large-scale open-pit coal mines, substantial quantities of coal resources are commonly left beneath the final slope due to constraints associated with slope stability, stripping ratio, and other factors [3]. Conventional end-wall mining can improve the recovery of these residual resources without markedly increasing the stripping volume [4]. However, the stability of mining entries, roof strata, and retained coal pillars remains a key factor limiting its wider application [5]. Backfill mining provides a promising technical approach to addressing these challenges [6]. This method can control roof deformation, reduce disturbance to the surrounding rock, improve the stress state of coal pillars, and promote the resource utilization of solid mining wastes [6,7]. A typical end-slope mining scenario and the distribution of residual resources beneath the final pit wall are illustrated in Figure 1.

Cemented backfill materials prepared from tailings, coal gangue, fly ash, slag, and other industrial by-products have been extensively investigated in both underground and open-pit mining engineering [8,9]. Such materials can consume large quantities of solid wastes and reduce the environmental pressure caused by surface stockpiling [10,11]. For open-pit coal mines, the use of overburden materials and fly ash as raw materials for backfill is particularly attractive, as these two materials are typically generated in large quantities near the mining area [12,13]. Their local availability helps reduce transportation costs, decrease cement consumption, and promote the construction of green and low-carbon mines [14,15].

Nevertheless, the application of conventional cemented backfill materials in end-wall mining entries still has certain limitations [16]. End-wall entries are generally characterized by narrow and elongated spaces, difficult backfilling operations, and challenges in achieving complete filling [17]. During transportation, deposition, and hardening, ordinary cemented backfill slurry may undergo bleeding, settlement, or drying shrinkage, which can easily lead to the formation of voids or separations between the hardened backfill body and the roof [18,19]. Once effective contact between the backfill body and the roof cannot be achieved, its actual supporting capacity will be significantly weakened, even if the backfill itself possesses high compressive strength [20,21]. Therefore, for end-wall backfill mining, the backfill material should simultaneously exhibit sufficient flowability for pipeline transportation, expansive capacity for active roof contact, and mechanical strength to meet load-bearing requirements [22,23].

Expansive backfill materials offer a possible solution to the insufficient roof-contact problem associated with conventional backfill materials [24]. By introducing expansive components into cementitious slurry, controlled volumetric expansion can be generated during hydration or the early hardening stage [25]. Compared with solid-phase expansion, gas-phase expansion can produce more pronounced early-stage volume increase and may therefore be more suitable for active roof contact in confined mining-entry spaces [26]. Hydrogen peroxide is a typical gas-generating expansive agent [27]. In an alkaline cementitious environment, hydrogen peroxide decomposes to release oxygen, forming a solid–liquid–gas three-phase slurry system. The generated bubbles increase slurry volume and enhance its ability to actively fill roof voids [28]. However, excessive gas generation may increase porosity, weaken the cemented structure, and reduce mechanical strength [29]. Therefore, balancing expansive capacity and mechanical performance is a key issue in the design of such materials [30].

In addition to mix proportion design, the mechanical behavior of hardened expansive backfill materials also requires further clarification [19,20]. To evaluate their load-bearing capacity and stability, it is necessary to investigate their uniaxial compressive deformation characteristics and shear strength parameters at different curing ages [21]. Moreover, the strength development mechanism of solid-waste-based expansive backfill materials is closely related to their microstructure [7,22]. Hydration products, pore structure, aggregate interlocking, and the distribution of gas-induced pores jointly determine their macroscopic mechanical response [28,31]. Therefore, microstructural observation is required to clarify how the material simultaneously achieves expansion and load-bearing functions [32,33].

To address these issues, this study developed a novel solid-waste-based expansive backfill material suitable for end-wall mining. Orthogonal experiments were conducted to investigate the effects of multiple mix proportion parameters on the physical and mechanical properties of the material, and the optimal mix proportion was determined based on its comprehensive performance. Subsequently, uniaxial compression and variable-angle shear tests were performed to evaluate the strength evolution, deformation characteristics, and failure response of the material under different stress states. In addition, scanning electron microscopy (SEM) was employed to elucidate the internal strengthening mechanisms of the material. The research findings can serve as a reference for the design of solid-waste-based expansive filling materials and their application in green and efficient end-mining operations.

## 2. Materials and Methods

### 2.1. Raw Materials

The expansive backfill material was prepared using mine overburden, ordinary Portland cement, fly ash and hydrogen peroxide. Mine overburden collected from the Heishan open-pit coal mine was used as the aggregate. The mineral composition content of the aggregate is shown in Table 1. To reduce the influence of particle-size effects and to obtain a stable aggregate gradation, the maximum particle size was controlled below 5 mm. The Talbot continuous grading theory was used to determine the aggregate gradation, and the grading index was set as 0.5. The mass fractions of particles in each size interval under this grading index are shown in Table 2.

P.O 42.5 ordinary Portland cement produced by Xinjiang Tianshan Cement Co., Ltd. was used as the primary binder. Class I fly ash produced by Henan Wuhu Environmental Protection Technology Co., Ltd. was used as a supplementary cementitious material to partially replace cement. The main chemical composition contents of cement and fly ash are shown in Table 3.

Hydrogen peroxide was used as the gas-generating expansive agent. A food-grade hydrogen peroxide solution with a mass concentration of 30% was adopted. In the alkaline cementitious slurry, hydrogen peroxide decomposes to release oxygen. The generated oxygen bubbles expand the slurry volume and enable active roof-contact during the early hardening stage.

### 2.2. Experimental Design and Specimen Preparation

An orthogonal experimental design was employed to investigate the effects of multiple interacting factors on the physical and mechanical properties of the material. The aggregate-to-binder ratio, fly ash dosage, and hydrogen peroxide dosage were selected as the experimental factors, while the flowability and expansion ratio of the filling slurry, together with the uniaxial compressive strength of the backfill material, were adopted as the evaluation indices. Before constructing the orthogonal array, preliminary single-factor experiments were carried out for each variable. Based on the response data obtained from these pretests, three representative levels were selected within the investigated effective range for each factor, corresponding to low, intermediate, and high values that produced distinguishable changes in flowability, expansion ratio, and compressive strength. The resulting levels are presented in Table 4. A water-to-binder ratio of 1:1 was maintained for all mixtures, where the binder mass is the total mass of cement and fly ash; therefore, the mass of separately added mixing water was equal to the total binder mass. The water contained in the 30% hydrogen peroxide solution was not included in the 1:1 water-to-binder ratio, because the hydrogen peroxide solution was dosed separately as an expansive-agent solution relative to the total binder mass. The fly ash dosage was defined as the mass percentage of fly ash in the total binder, and the hydrogen peroxide dosage was defined as the mass percentage of hydrogen peroxide solution relative to the total binder mass.

Based on the number of experimental factors and their levels, an L9 (3^4) orthogonal array was employed to arrange the experiments. Three columns were assigned to the three influencing factors, while the remaining column was designated as an error column to improve the reliability of the range analysis and analysis of variance (ANOVA). The orthogonal experimental design is presented in Table 5. It should be noted that, because the three factors vary simultaneously in the orthogonal array, the level-mean response of each factor represents an averaged marginal effect over the levels of the other factors rather than an isolated single-factor response. The orthogonal design is therefore used here primarily to rank the relative influence of the factors and to identify suitable level combinations, rather than to establish a continuous monotonic relationship for an individual component.

**Figure 2:**
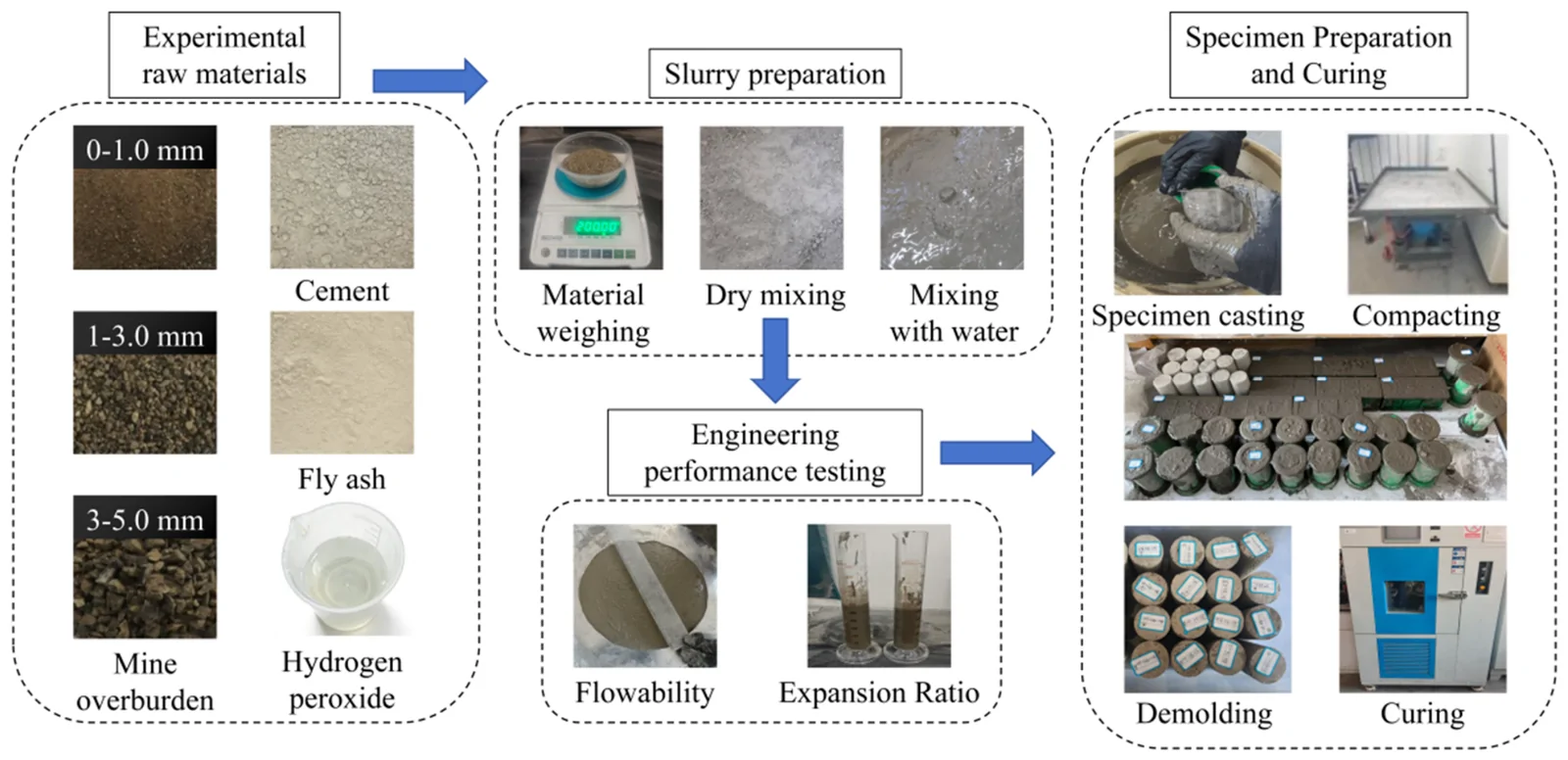
Preparation procedure for expansive backfill material specimens.

The preparation procedure for specimens of the expansive backfill material was conducted in accordance with the relevant provisions of the Test Methods for Paste Backfill Materials in Coal Mines (NB/T 51070-2017) [34]. The procedure comprised five steps: proportioning, mixing, mold casting, demolding, and curing, as illustrated in Figure 2. According to the designed mixture proportions, mine overburden, cement, fly ash, water and hydrogen peroxide were weighed separately. The solid materials, including mine overburden, cement and fly ash, were first added into a JJ-5 cement mortar mixer and dry-mixed for 3 min to ensure uniform dispersion. Water was then added gradually, and the mixture was wet-mixed for 5 min. Finally, hydrogen peroxide was added into the fresh slurry, followed by another 3 min of mixing to ensure that the expansive agent was uniformly distributed in the slurry. Immediately after mixing, the fresh slurry was used for fluidity and expansion-ratio tests.

For mechanical tests, the fresh slurry was poured into lubricated molds. Cylindrical specimens with a diameter of 50 mm and a height of 100 mm were prepared for uniaxial compression tests. Cubic specimens with dimensions of 50 mm × 50 mm × 50 mm were prepared for variable-angle shear tests. After casting, the molds were vibrated for 10 s to reduce large entrapped air voids and improve specimen uniformity. The specimens were covered with plastic film to prevent moisture evaporation. After 1 h, the expanded part above the mold surface was removed and the specimen surface was leveled. The specimens were de-molded after 24 h and then cured in a standard curing chamber at a temperature of approximately 20 °C and a relative humidity above 90% until the designed testing ages.

### 2.3. Testing Methods

The fluidity of the fresh backfill slurry was measured using a cement mortar flow table according to GB/T 2419–2005 [35]. The expansion ratio was measured using a 500 mL graduated cylinder. The freshly mixed slurry was poured into the cylinder and gently vibrated to ensure uniform filling, after which the slurry surface was read directly against the cylinder graduations. The initial slurry volume L_0_ (mL) was recorded immediately after casting. The cylinder was then sealed with plastic film to prevent moisture loss. Since the slurry containing hydrogen peroxide showed significant expansion within the first hour and little further expansion afterwards, the slurry volume L_x_ (mL) was recorded after 1 h. Thus, L_0_ and L_x_ denote direct volume readings from the graduated cylinder rather than specimen heights. The expansion ratio was calculated using the following equation:

(1) $$E_{x}=\frac{L_{x}-L_{0}}{L_{0}}\times 100\%$$

where E_x_ is the expansion ratio of the slurry, L_x_ is the slurry volume reading after 1 h of expansion (mL), and L_0_ is the initial slurry volume reading immediately after casting (mL).

**Figure 3:**
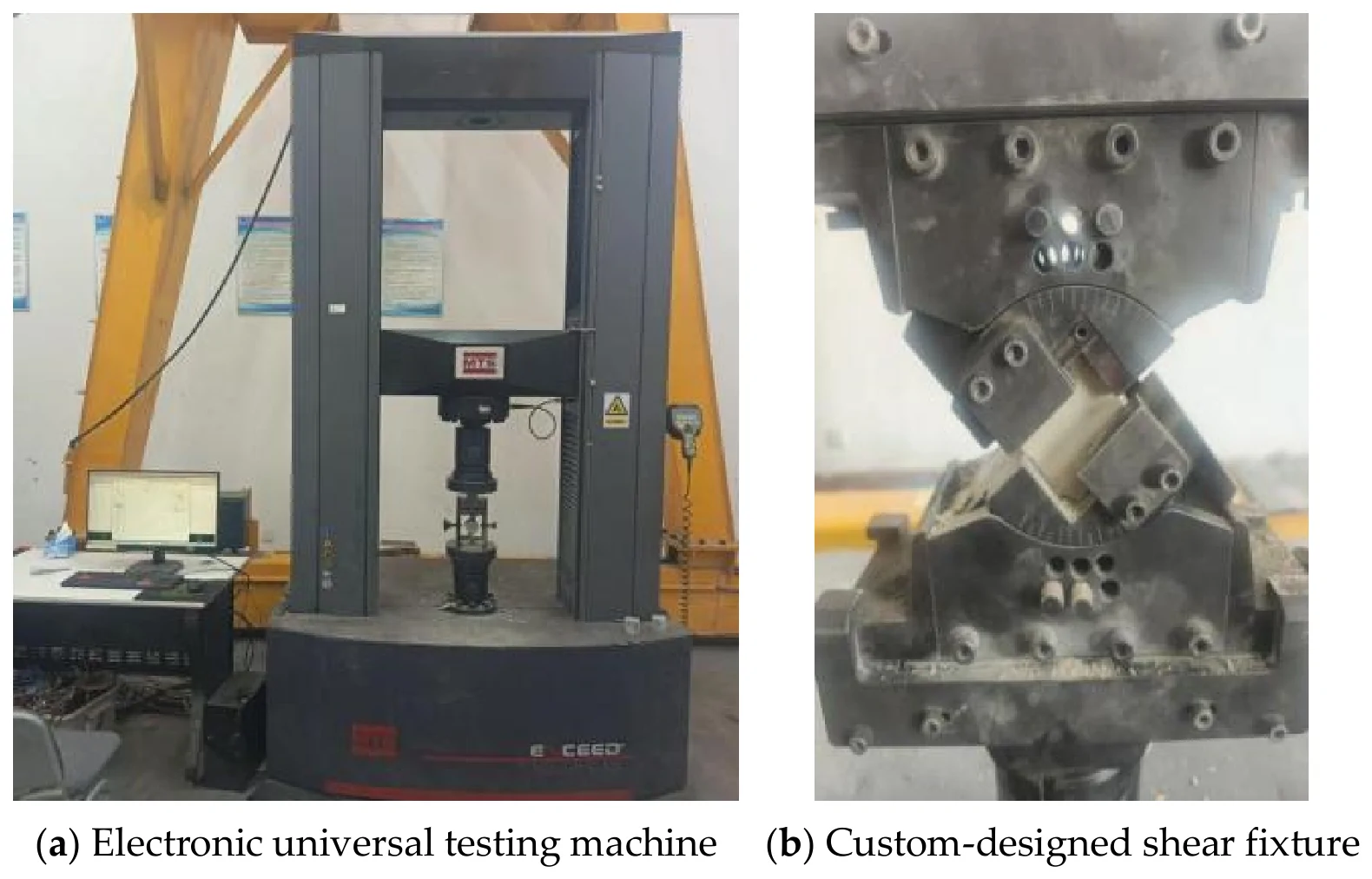
Equipment for mechanical property testing.

Mechanical property tests included uniaxial compression and variable-angle shear tests. The testing apparatus is shown in Figure 3. The uniaxial compression tests were conducted under displacement-controlled loading, with the loading rate uniformly set at 0.5 mm/min. During loading, the axial load and corresponding axial displacement were recorded by the testing system. Engineering stress and strain were calculated from the measured load and displacement using the initial specimen geometry. The stress–strain curves were generated from the raw experimental stress–strain data recorded by the testing system without curve fitting or manual alteration. For the variable-angle shear tests, five shear angles, namely 40°, 45°, 50°, 55°, and 60°, were selected. Three parallel tests were performed at each angle, and the axial load was applied at a constant rate of 0.5 MPa/s. For each shear specimen, the failure load obtained at the prescribed fixture angle was converted independently into the corresponding normal and shear stresses on the shear plane. The three parallel results at each angle were retained as individual stress pairs rather than averaged before fitting. In the variable-angle shear configuration, the cubic specimen was positioned in the custom fixture so that loading acted across a prescribed inclined shear plane. Conceptually, the peak axial load P was resolved into a component N normal to the prescribed plane and a component T tangential to it according to the fixture geometry; the corresponding stresses were calculated as σ = N/A and τ = T/A, where A is the nominal area of the shear plane. The five reported shear angles refer to the five prescribed fixture inclinations used to change the relative normal and tangential components of the applied load.

Because gas-generating expansion introduced pores and microvoids into the hardened backfill, the initial nonlinear portion of the stress–strain response was treated as a pore-compaction stage rather than as an elastic deformation stage. Accordingly, the tangent slope close to zero strain should not be interpreted as the elastic modulus. The elastic modulus values were calculated automatically from the recorded test data by the built-in control software of the testing machine according to its internal calculation routine, rather than being manually determined from the visually apparent tangent slope at zero strain.

## 3. Results and Discussion

### 3.1. Orthogonal Experimental Results of Expansive Backfill Material

**Figure 4:**
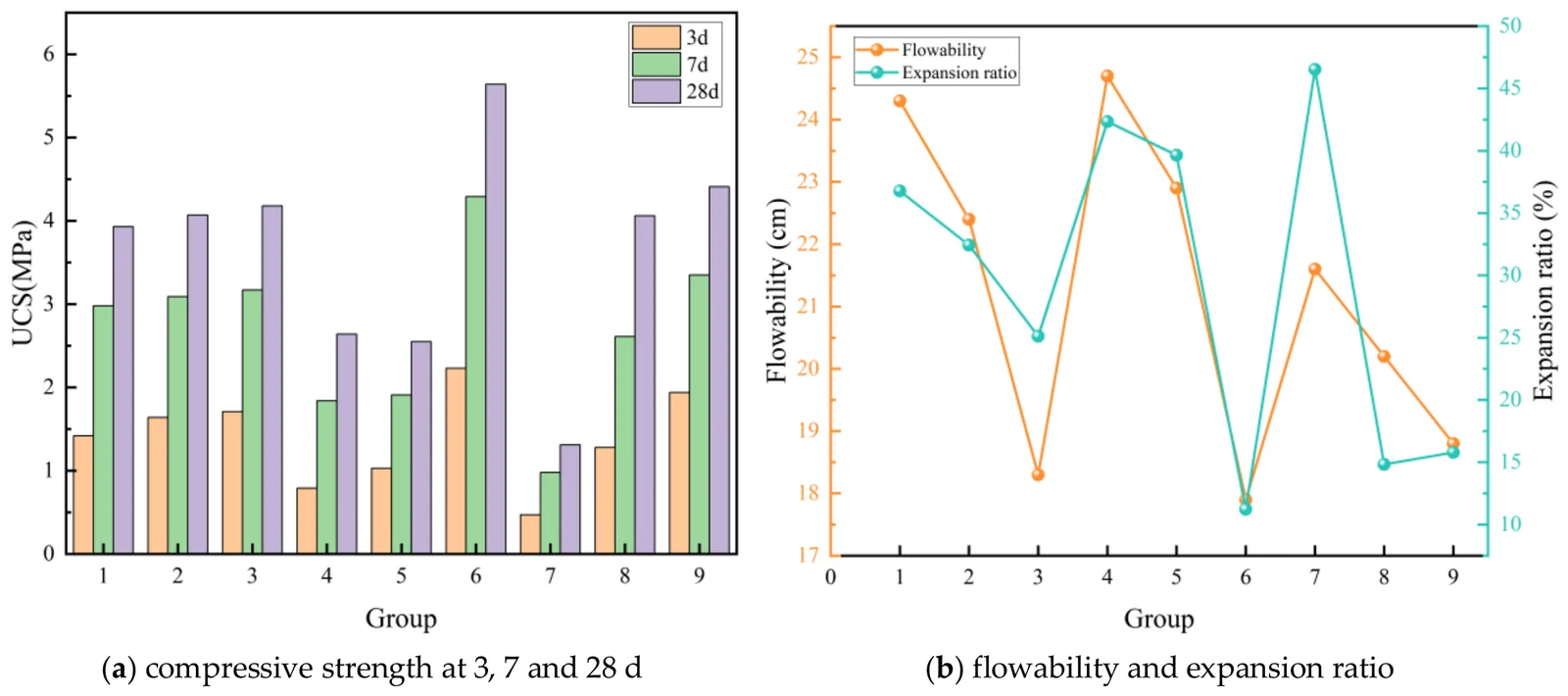
Orthogonal experimental results.

The orthogonal experimental results of the expansive backfill mixtures are presented in Figure 4. The flowability of the fresh mixtures ranged from 17.9 cm to 24.7 cm, indicating that the prepared slurries maintained a certain flow capacity under different mixture proportions. The expansion ratio varied more significantly, ranging from 11.23% to 46.52%, which confirms that hydrogen peroxide effectively introduced active volumetric expansion into the slurry system.

The compressive strength increased with curing age for all mixtures. The 3 d, 7 d and 28 d compressive strengths were 0.47–2.23 MPa, 0.98–4.29 MPa and 1.31–5.64 MPa, respectively. The mixture containing 40% fly ash, an aggregate-to-binder ratio of 6:1, and 5% hydrogen peroxide exhibited the highest compressive strength at all curing ages. In contrast, the mixture containing 50% fly ash, an aggregate-to-binder ratio of 2:1, and 20% hydrogen peroxide showed the highest expansion ratio but the lowest compressive strength. This direct compositional contrast indicates that a higher expansion ratio does not necessarily correspond to better overall performance, because the combination of a low aggregate-to-binder ratio and a high hydrogen peroxide dosage promotes gas expansion but can also reduce the compactness of the hardened matrix.

The orthogonal results are supported by the measured contrasts in Figure 4 rather than by a single qualitative assumption. Across the nine mixtures, the expansion ratio varied from 11.23% to 46.52%; whereas, the 28 d compressive strength varied from 1.31 MPa to 5.64 MPa. The high-expansion mixture combined 50% fly ash, an aggregate-to-binder ratio of 2:1, and 20% hydrogen peroxide, while the highest-strength mixture combined 40% fly ash, an aggregate-to-binder ratio of 6:1, and 5% hydrogen peroxide. These opposing responses demonstrate that workability, expansion behavior, and mechanical strength are coupled and cannot be maximized simultaneously. Therefore, a rational mixture design should balance slurry transportability, controlled expansion for roof contact, solid-waste utilization, and mechanical stability. Based on these measured differences, further range analysis and variance analysis were conducted to clarify the relative influence of each mixture parameter on the main performance indicators.

### 3.2. Influence of Mixture Parameters on Flowability, Expansion Ratio and 28 d Compressive Strength

**Figure 5:**
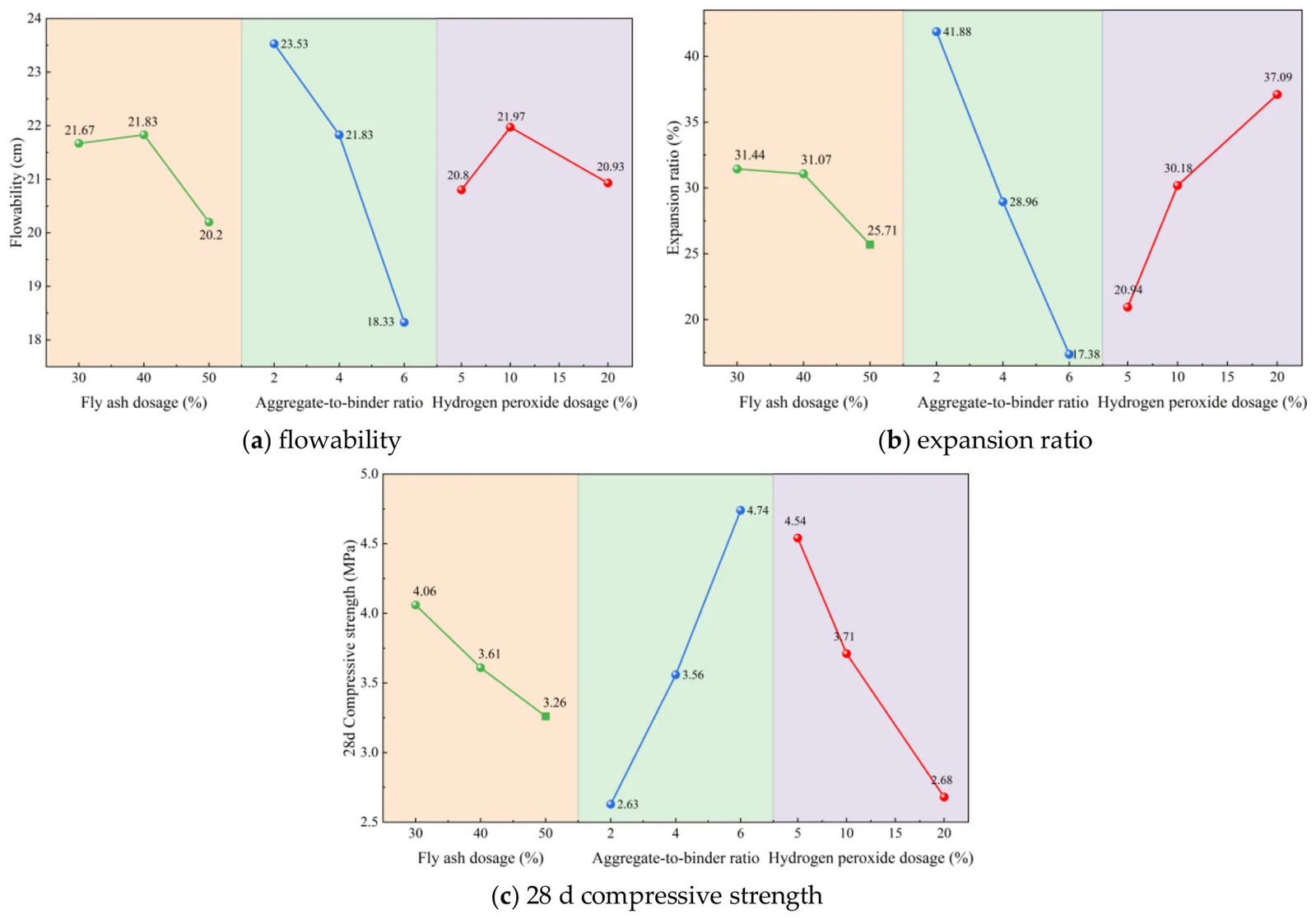
Effects of mixture parameters on the main performance indicators.

To further clarify the effects of the main mixture parameters on the performance of the expansive solid-waste-based backfill material, the level-mean values of flowability, expansion ratio and 28 d compressive strength were calculated based on the orthogonal experimental results. The corresponding factor-effect trends are shown in Figure 5.

#### 3.2.1. Effect of Mixture Parameters on Flowability

As shown in Figure 5a, the flowability of the backfill slurry was mainly affected by the aggregate-to-binder ratio. When the aggregate-to-binder ratio increased from 2:1 to 6:1, the average flowability decreased continuously from 23.53 cm to 18.33 cm. This is because increasing the aggregate-to-binder ratio decreases the relative proportion of cementitious slurry and free water in the mixture. Meanwhile, more solid particles increase internal friction and particle collision during flow, thereby reducing the mobility of the fresh slurry.

The influence of fly ash dosage on flowability was relatively weaker than that of the aggregate-to-binder ratio. The highest average flowability was obtained at a fly ash dosage of 40%. This result can be attributed to the morphological and filling effects of fly ash particles: an appropriate amount of fly ash can improve particle packing and reduce friction among solid particles, thereby enhancing slurry flowability. A similar beneficial effect of fly ash on the workability of cemented coal-gangue paste backfill was reported by Cheng et al. [12], who observed increased slump with increasing fly ash content, although the response remained dependent on the complete mixture composition. The hydrogen peroxide dosage also showed a non-monotonic influence on flowability. A moderate amount of hydrogen peroxide can introduce gas bubbles into the slurry, which may reduce the apparent density and improve short-term mobility. According to the factor-effect trend, the flowability-preferred combination was 40% fly ash, an aggregate-to-binder ratio of 2:1, and 10% hydrogen peroxide.

The non-monotonic trends of fly ash and hydrogen peroxide in Figure 5a should be interpreted in the context of the orthogonal design. Because the level means are averaged over different combinations of the other factors, the plotted response reflects the net result of several competing mechanisms. For fly ash, the moderate dosage of 40% can improve particle packing and reduce interparticle friction; whereas, this beneficial effect becomes weaker at 50%, where the increased proportion of fine particles alters particle interactions. For hydrogen peroxide, the 30% H_2_O_2_ solution is introduced in addition to the fixed mixing water and its contained water is not counted in the 1:1 water-to-binder ratio; thus, increasing its dosage increases the total liquid introduced into the mixture, while the simultaneously intensified oxygen generation changes the apparent density and the solid–liquid–gas structure of the fresh slurry. These effects can partially offset one another and lead to the observed non-monotonic response. Accordingly, the fly ash and hydrogen-peroxide trends in Figure 5a should not be regarded as isolated single-factor monotonic relationships, but as coupled marginal responses within the selected orthogonal matrix.

#### 3.2.2. Effect of Mixture Parameters on Expansion Ratio

The factor-effect trends of expansion ratio are shown in Figure 5b. The aggregate-to-binder ratio had the most pronounced effect on the expansion behavior of the fresh slurry. As the aggregate-to-binder ratio increased from 2:1 to 6:1, the average expansion ratio decreased sharply from 41.88% to 17.38%. This indicates that a higher aggregate content was unfavorable for gas-generating expansion. When the binder content is relatively high, the alkaline cementitious slurry provides a more suitable environment for hydrogen peroxide decomposition and gas-bubble stabilization. In contrast, increasing the aggregate proportion reduces slurry continuity and weakens the ability of the matrix to retain bubbles, resulting in a lower expansion ratio.

Hydrogen peroxide dosage was another important factor controlling the expansion ratio. In the alkaline environment of the cement-based system, hydrogen peroxide decomposes and releases oxygen bubbles, which increase the slurry volume and contribute to active roof-contact potential. However, although a higher hydrogen peroxide dosage increases expansion, it may also introduce excessive pores into the hardened backfill, which can adversely affect mechanical strength. The gas-generation and slurry-expansion behavior of H_2_O_2_-foamed cementitious systems has similarly been emphasized by Xie et al. [24]. Compared with the aggregate-to-binder ratio and hydrogen peroxide dosage, fly ash dosage had a weaker influence on the expansion ratio. The expansion-preferred combination was 30% fly ash, an aggregate-to-binder ratio of 2:1, and 20% hydrogen peroxide.

#### 3.2.3. Effect of Mixture Parameters on 28 d Compressive Strength

Figure 5c shows the effects of the three mixture parameters on the 28 d compressive strength. The trend of compressive strength was clearly different from those of flowability and expansion ratio. The 28 d compressive strength increased significantly with increasing aggregate-to-binder ratio. When the aggregate-to-binder ratio increased from 2:1 to 6:1, the average 28 d compressive strength increased from 2.63 MPa to 4.74 MPa. This result indicates that the aggregate framework played an important role in the long-term load-bearing capacity of the hardened backfill. A higher aggregate proportion enhanced the skeleton-support effect and improved the overall compressive resistance of the material.

In contrast, increasing fly ash dosage reduced the 28 d compressive strength. Although fly ash can improve slurry workability and participate in pozzolanic reactions, excessive replacement of cement reduces the amount of primary hydration products available during strength development and weakens the early-to-intermediate cementitious framework. A comparable reduction or delay in strength development with increasing fly ash replacement has been reported for cemented paste backfill by Behera et al. [8], who linked the macroscopic strength response to binder dosage and microstructural evolution. The present result therefore reflects the balance between the workability and solid-waste-utilization benefits of fly ash and the reduction in Portland-cement-derived bonding when the replacement level becomes high.

Hydrogen peroxide dosage also had a negative effect on 28 d compressive strength. This decline can be explained by the pore-forming effect of gas generation. A moderate amount of bubbles can provide the desired expansion for active roof contact, but excessive hydrogen peroxide produces more gas-induced pores and weakens the compactness of the hardened matrix. Similar competition between entrained gas or pore formation and mechanical performance has been reported for air-entrained cemented backfill and H_2_O_2_-foamed cementitious materials [23,24,30]. Therefore, the material shows a typical trade-off between expansion performance and mechanical strength. The strength-preferred combination was 30% fly ash, an aggregate-to-binder ratio of 6:1, and 5% hydrogen peroxide.

### 3.3. Engineering-Oriented Multi-Index Selection of Mix Proportion

The range analysis showed that the preferred level combinations differed among flowability, expansion ratio, and 28 d compressive strength. This means that the final mixture cannot be selected from a single performance index. In the present study, the term comprehensive selection refers to an engineering-oriented trade-off among several practical requirements rather than to a formal mathematical optimization.

For end-slope mining, the backfill slurry should have sufficient flowability to ensure transportation and diffusion in narrow mined-out openings, controlled expansion to compensate for shrinkage and improve roof-contact performance, and adequate long-term strength to form an effective load-bearing structure. Solid-waste utilization and binder consumption are also important engineering considerations. No numerical weighting coefficients or objective function were assigned to these indices in this study; instead, they were treated as concurrent engineering constraints and considered together when selecting the final mixture.

The single-index results demonstrate the basis of this engineering trade-off. An aggregate-to-binder ratio of 2:1 improved flowability and expansion but increased binder consumption and reduced mine-overburden utilization; whereas, a ratio of 6:1 enhanced 28 d compressive strength but reduced flowability and expansion capacity. Therefore, the intermediate ratio of 4:1 was selected. Similarly, 40% fly ash was selected to balance cement replacement, solid-waste utilization, workability, and strength development. For hydrogen peroxide, 10% was selected because it provided effective gas-generating expansion while avoiding the greater pore formation and strength reduction associated with 20%. The comparison of these single-index preferences and their engineering limitations is summarized in Table 6.

Based on the above engineering-oriented multi-index trade-off, the selected comprehensive mixture contained 40% fly ash, an aggregate-to-binder ratio of 4:1, and 10% hydrogen peroxide (A2B2C2). As summarized in Table 6, the preferred combinations for flowability, expansion ratio, and 28 d compressive strength were different and each involved an engineering limitation. The selected A2B2C2 mixture therefore does not mathematically maximize a weighted objective function or any single performance index; rather, it represents a practical compromise among slurry transportability, active roof-contact potential, long-term bearing capacity, solid-waste utilization, and binder economy. For consistency with the remainder of the manuscript, this engineering-selected composition is hereafter referred to as the optimized mixture.

Unless otherwise specified, the subsequent uniaxial compression, variable-angle shear and SEM tests were all conducted using this optimized A2B2C2 mixture; they were not comparisons among the nine orthogonal mixtures.

### 3.4. Age-Dependent Uniaxial Compressive Behavior of the Optimized Backfill

**Figure 6:**
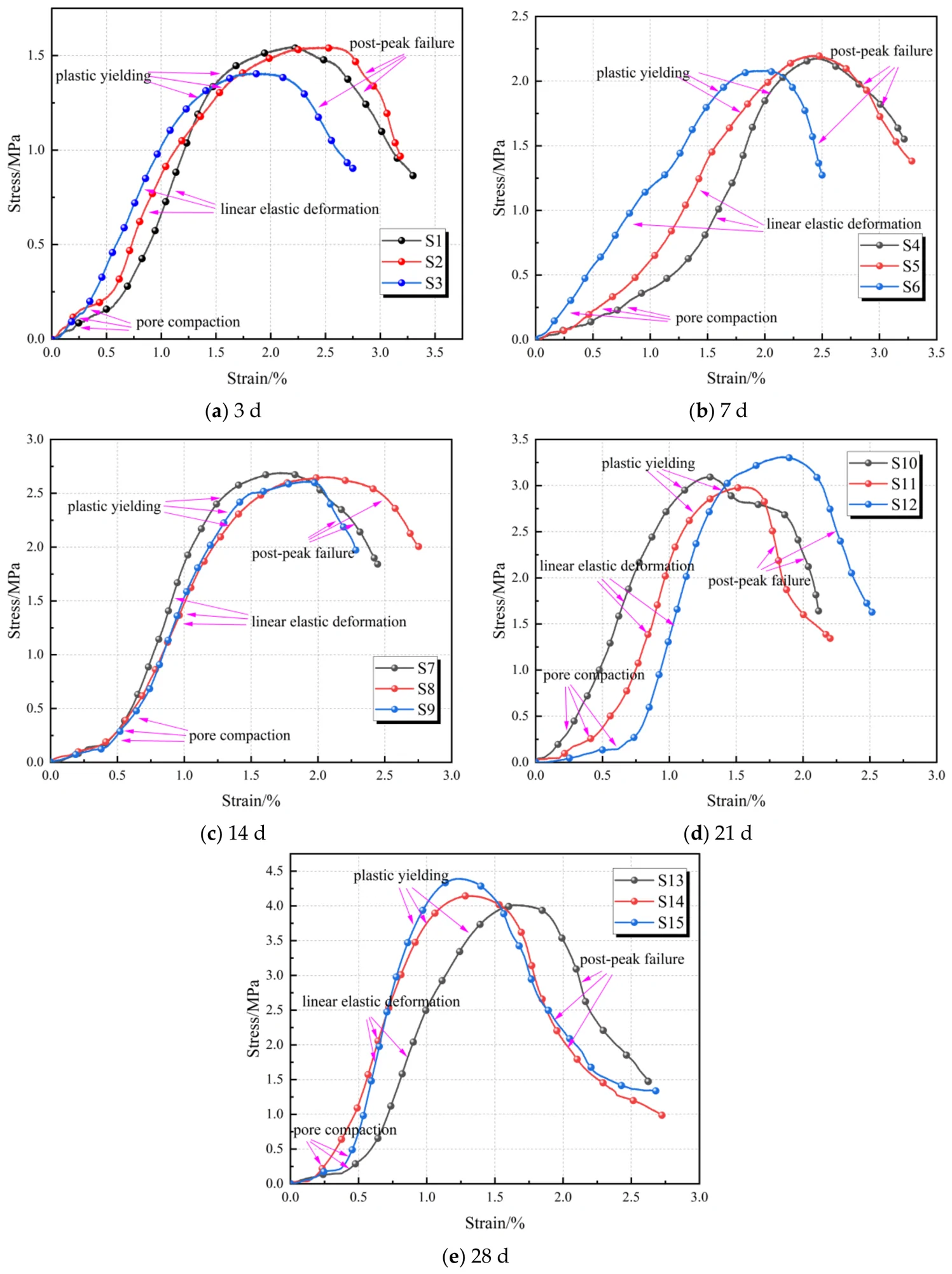
Experimental uniaxial stress–strain curves of the optimized A2B2C2 backfill at different curing ages: (**a**) 3 d (S1–S3); (**b**) 7 d (S4–S6); (**c**) 14 d (S7–S9); (**d**) 21 d (S10–S12); and (**e**) 28 d (S13–S15). S1–S15 denote individual parallel specimens rather than different mixture proportions. The initial nonlinear segment primarily represents pore compaction and should not be interpreted as the elastic modulus.

**Figure 7:**
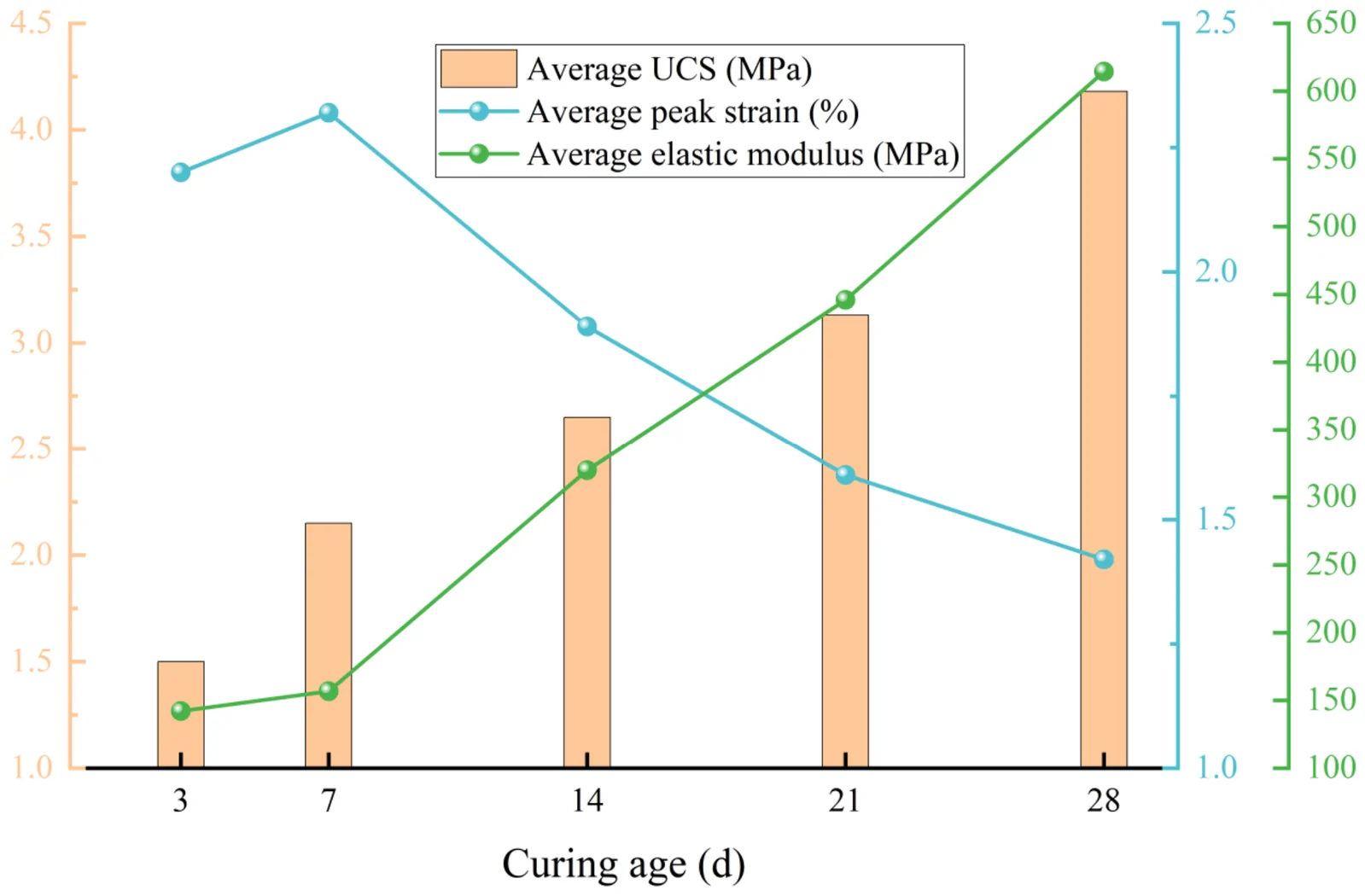
Mechanical parameters of the optimized backfill under uniaxial compression.

Uniaxial compression tests were conducted on the optimized backfill specimens at curing ages of 3, 7, 14, 21 and 28 d. The stress–strain curves are shown in Figure 6, and the corresponding mechanical parameters are shown in Figure 7. All specimens in Figure 6 were prepared with the optimized A2B2C2 composition. The labels S1–S15 are sequential specimen identifiers for the three parallel tests at each curing age (S1–S3, 3 d; S4–S6, 7 d; S7–S9, 14 d; S10–S12, 21 d; and S13–S15, 28 d), and do not represent additional mixture designs.

As shown in Figure 6, the uniaxial stress–strain curves of the optimized backfill at different curing ages exhibit four characteristic deformation stages: pore compaction, approximately linear elastic deformation, plastic yielding, and post-peak failure. It should be emphasized that the tangent slope immediately adjacent to zero strain does not represent the elastic modulus of the backfill.

During the initial loading stage, the curves exhibit a concave-upward response. Hydrogen-peroxide-induced gas generation leaves a certain amount of pores and microvoids in the hardened matrix. Under initial compression, these defects are progressively closed, accompanied by local particle rearrangement. Minor differences in the initial contact between the specimen end surfaces and the loading platens may also contribute to the deformation recorded at very low stress levels. Therefore, the apparent slope near zero strain reflects the combined effects of pore closure, particle rearrangement, and initial contact adjustment rather than the intrinsic elastic stiffness of the material. The relatively large differences in this initial segment among specimens should consequently not be interpreted directly as equivalent differences in elastic modulus.

After the pore-compaction stage, the specimens enter an approximately linear deformation region, whose slope characterizes the effective elastic stiffness. With increasing curing age, continued cement hydration and the pozzolanic reaction of fly ash generate additional cementitious products, which progressively fill internal pores, strengthen the aggregate–matrix interfaces, and improve the continuity of the load-bearing skeleton. The material therefore evolves from a relatively porous and compliant early-age structure toward a denser and stiffer hardened structure. After the approximately linear stage, progressive initiation and propagation of internal cracks cause the curves to deviate from linearity and enter the plastic-yielding stage. Once the peak stress is reached, crack coalescence and macroscopic fracture lead to a gradual decrease in load-bearing capacity. Thus, the age-dependent evolution of the stress–strain response results from the combined effects of the initial pore structure and progressive hydration-induced matrix densification. This interpretation is consistent with previous cemented-backfill studies in which curing age and the progressive formation of hydration products were identified as major controls on strength development and matrix densification [8,12].

As shown in Figure 7, the strength increased rapidly from 3 d to 7 d, then grew more slowly from 7 d to 21 d, and increased again between 21 d and 28 d. This staged strength-growth behavior reflects the combined effect of gas-induced pore formation and hydration-induced matrix densification. In the early stage, cement hydration rapidly formed initial bonding strength, but the pores introduced by hydrogen peroxide limited the compactness of the matrix. With extended curing, hydration products gradually filled pores and improved the bonding between aggregate particles, leading to further strength enhancement.

The elastic modulus values shown in Figure 7 were calculated automatically by the testing machine’s built-in control software from the recorded test data and should not be equated with the visually apparent slope of the initial pore-compaction segment in Figure 6. The age-dependent increase in the reported modulus is consistent with progressive stiffening of the material: continued hydration not only enhanced the ultimate load-bearing capacity but also strengthened the internal aggregate–cementitious skeleton. In contrast, the average peak strain generally decreased after 7 d, from 2.32% at 7 d to 1.42% at 28 d, indicating that the material gradually evolved from a relatively deformable early-age structure into a denser and stiffer hardened structure.

**Figure 8:**
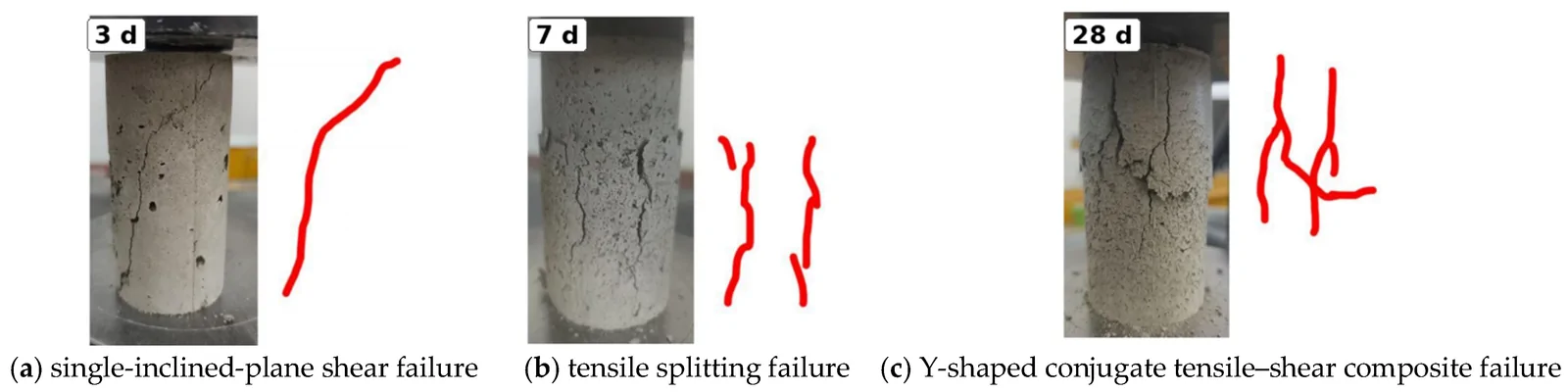
Representative failure patterns of the optimized backfill under uniaxial compression at different curing ages: (**a**) 3 d, single-inclined-plane shear failure; (**b**) 7 d, tensile splitting failure; and (**c**) 28 d, Y-shaped tensile–shear composite failure.

The age-dependent evolution of the failure patterns shown in Figure 8 can be interpreted from stress transformation and the progressive development of the cementitious structure. Under an ideal uniaxial compressive stress state, the major principal stress is approximately equal to the applied axial stress (σ1 = σa); whereas, the minor principal stress approaches zero (σ3 ≈ 0). For an arbitrary plane, the transformed normal and shear stresses can be expressed as σn = (σ1 + σ3)/2 + (σ1 − σ3)cos(2θ)/2 and τ = (σ1 − σ3)sin(2θ)/2, respectively. The corresponding Mohr circle indicates that substantial shear stress develops on inclined planes, with the maximum shear stress occurring on planes oriented at approximately 45° to the principal planes under the ideal uniaxial condition.

For the optimized expansive backfill, the variable-angle shear tests discussed in Section 3.5 show that the shear behavior follows the Mohr–Coulomb criterion, τf = c + σn tanφ, with a cohesion c of 1.61 MPa and an internal friction angle φ of 30.13°. According to this framework, the theoretical shear-failure plane is inclined at approximately 45° + φ/2 = 60.1° relative to the major principal plane, corresponding to approximately 29.9° relative to the axial loading direction. The occurrence of an inclined macroscopic shear plane in the relatively weak specimens is therefore mechanically consistent with the frictional–cohesive characteristics of the backfill, although the actual crack orientation can deviate from the theoretical value because of material heterogeneity and boundary effects.

The actual stress field within a cylindrical specimen is not perfectly uniaxial. Friction between the specimen ends and the loading platens restrains lateral expansion near the end surfaces and generates transverse compressive stress, producing a locally confined stress state. This confinement decreases away from the ends, so the stress state varies along the specimen height. In addition, gas-induced pores, irregular overburden particles, and heterogeneous aggregate–matrix interfaces cause local stress concentrations. These effects produce a non-uniform internal stress field and provide preferential sites for the initiation of tensile and shear microcracks. The interpretation presented here is based on the observed fracture morphology and classical mechanics; no full-field internal stress measurement was performed in the present tests.

At 3 d, cement hydration is still incomplete and the bonding between aggregate particles and the cementitious matrix is comparatively weak. Once the stress state reaches the shear-strength envelope, deformation tends to localize rapidly along a dominant inclined weak plane, resulting in the single-inclined-plane shear failure shown in Figure 8a. By 7 d, continued hydration and the pozzolanic reaction of fly ash have progressively strengthened the aggregate–matrix interfaces and increased the stiffness and compressive strength of the specimen. Premature localization along a single weak plane is therefore suppressed, while heterogeneous lateral deformation and local stress concentrations can promote tensile microcracks approximately parallel to the loading direction, leading to the tensile splitting observed in Figure 8b. At 28 d, the stronger and more continuous cementitious skeleton allows multiple tensile and shear cracks to propagate and interact before final failure. Their coalescence produces the branched crack paths and Y-shaped tensile–shear composite failure shown in Figure 8c. Therefore, the transition from inclined shear failure at 3 d to tensile splitting at 7 d and tensile–shear composite failure at 28 d reflects the combined effects of hydration-induced structural strengthening, material heterogeneity, stress redistribution, and end-restraint effects during uniaxial compression.

### 3.5. Shear Strength Parameters of the Optimized Backfill

**Figure 9:**
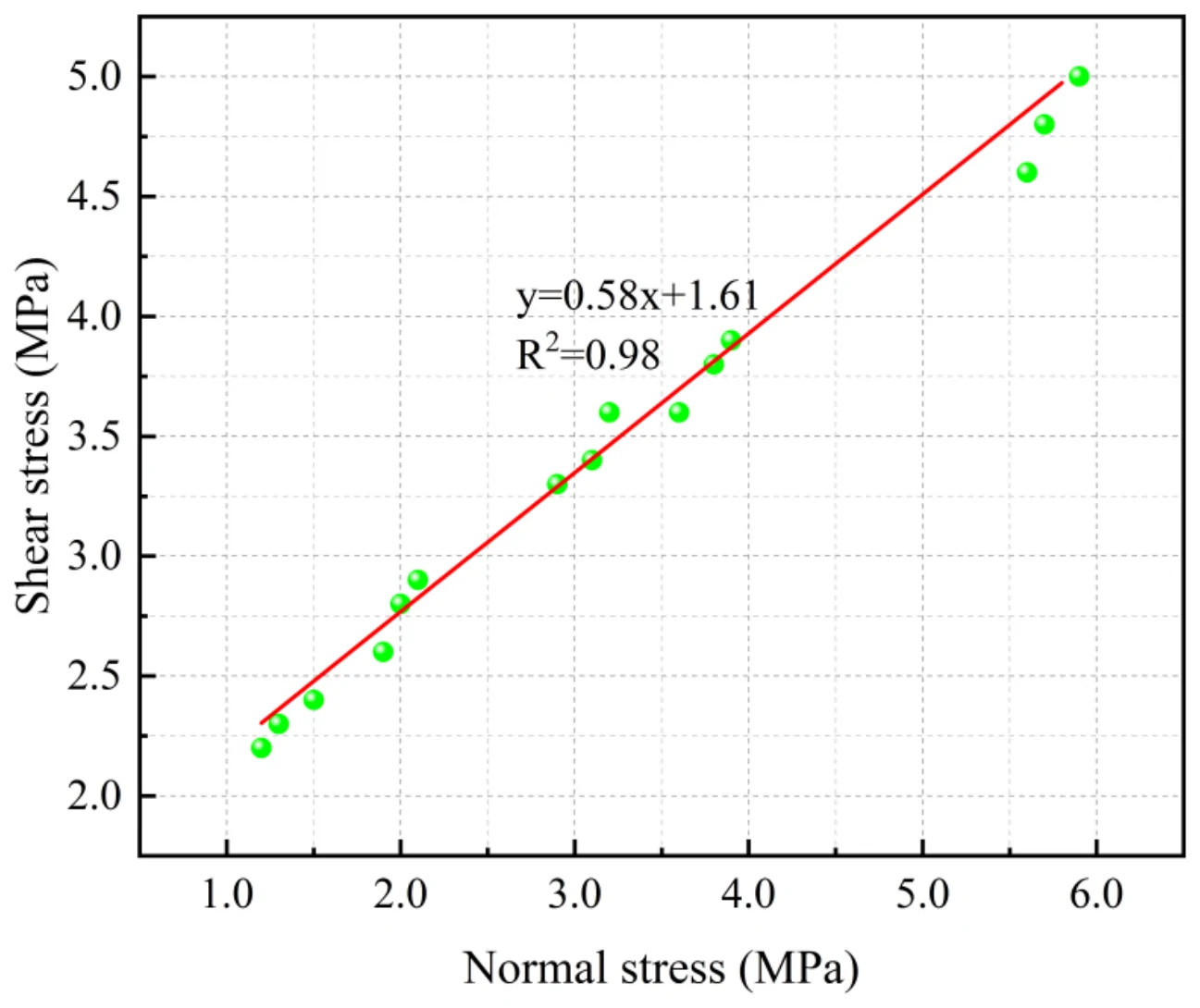
Linear fitting curve of shear strength for the optimized backfill (The green circles represent the experimental data, and the red line represents the linear regression).

Since shear failure was frequently observed in the uniaxial compression tests, the shear resistance of the material is an important parameter for assessing its stability and load-bearing capacity in end-slope mining. Combined with the Mohr–Coulomb strength criterion, the variable-angle shear test data were linearly fitted to obtain the shear strength line of the expansive backfill, as shown in Figure 9. Each point in Figure 9 represents the converted normal–shear stress pair from one individual specimen. Thus, three points correspond to each of the five fixture angles, and the regression was performed using all 15 individual test results rather than angle-wise averaged values. This presentation retains the within-angle scatter of the parallel tests while allowing the overall Mohr–Coulomb trend to be evaluated.

The fitting equation can be expressed as:

(2) τ=0.58σ+1.61

with a coefficient of determination R^2^ = 0.98. This indicates that the Mohr–Coulomb criterion can reasonably describe the shear behavior of the optimized expansive backfill. According to the intercept and slope of the fitted line, the cohesion and internal friction angle were determined as 1.61 MPa and 30.13°, respectively.

The cohesion mainly reflects the cementitious bonding provided by hydration products, while the internal friction angle is closely related to the rough surface and interlocking effect of mine overburden particles. Although hydrogen peroxide introduced pores into the matrix to provide expansion capacity, the aggregate skeleton and cementitious bonding still enabled the hardened backfill to maintain good shear resistance. Therefore, the optimized backfill can not only provide active roof-contact expansion but also resist shear deformation under constrained loading conditions. The interpretation that cementitious bonding controls cohesion while particle roughness and interlocking contribute to frictional resistance is also consistent with the broader mechanical understanding of cemented backfill and backfill–rock shear systems [19,20].

### 3.6. Microstructural Mechanism of Expansion and Strength Formation

**Figure 10:**
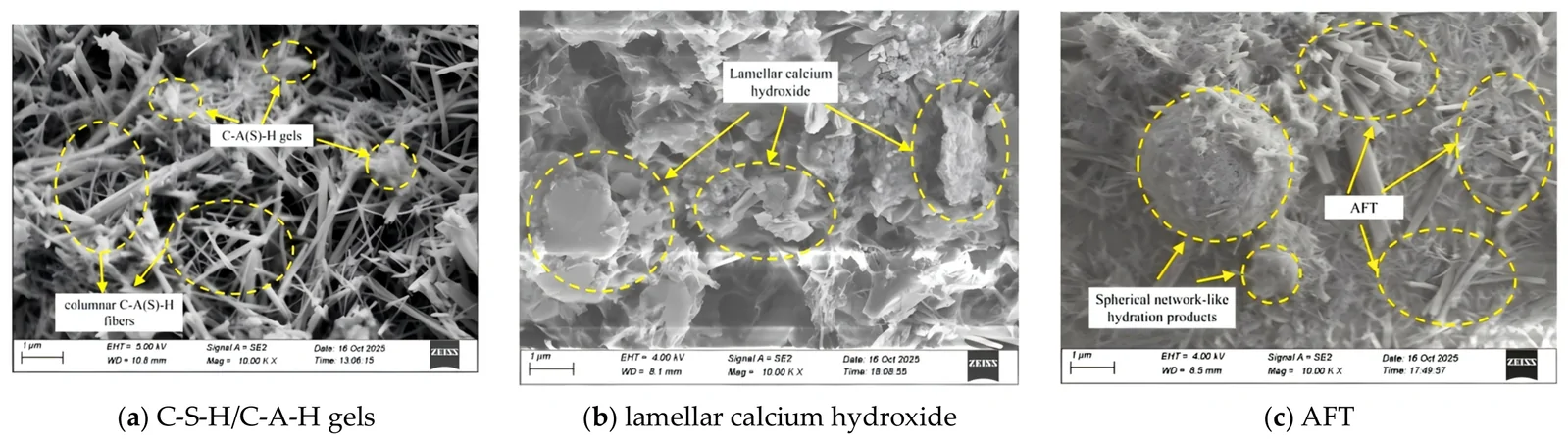
SEM images of the optimized expansive backfill.

SEM observation was conducted to reveal the microstructural characteristics and strengthening mechanism of the optimized expansive backfill. The SEM images are shown in Figure 10. Various hydration products with flocculent, fibrous, needle-like and lamellar morphologies can be observed inside the hardened matrix.

As shown in Figure 10a, a large amount of flocculent and fibrous gel-like products was distributed around the aggregate particles and pore walls. These products were mainly C-S-H and C-A-H gels formed by cement hydration and the pozzolanic reaction of fly ash. During hydration, active SiO_2_ and Al_2_O_3_ in fly ash gradually dissolved in the alkaline pore solution and reacted with Ca^2+^ and OH^-^ released from cement hydration, producing additional cementitious gels. These gels filled internal pores, bonded adjacent particles and improved the compactness of the backfill matrix. Similar microstructural assemblages involving C-S-H, portlandite, and ettringite have been reported in fly ash-modified cemented paste backfill and were associated with curing-related strength development [8].

Figure 10b shows lamellar calcium hydroxide crystals generated during cement hydration. Although calcium hydroxide itself contributes limited strength, it provides an alkaline environment that promotes the activation of fly ash. Meanwhile, needle-like ettringite can be observed in Figure 10c. The ettringite crystals were mainly distributed near particle contact zones and pore walls, where they helped fill voids and bridge particles. The coexistence of C-S-H gel, C-A-H gel, calcium hydroxide and ettringite indicates that the cement-fly ash binder system formed a continuous cementitious network during curing.

The mine overburden particles acted as the main load-bearing skeleton of the backfill. Due to their irregular shape and rough surface, these particles provided mechanical interlocking and frictional resistance. Hydration products further wrapped and bonded the aggregate particles, forming a relatively stable aggregate–matrix structure. This explains the continuous increase in compressive strength and elastic modulus with curing age, as well as the favorable shear resistance obtained in the variable-angle shear tests.

Hydrogen peroxide played a different but equally important role in the microstructure. In the alkaline cementitious slurry, hydrogen peroxide decomposed and released oxygen bubbles, which caused early-age volume expansion. These bubbles enabled the slurry to compensate for shrinkage and improve active roof-contact potential. However, excessive gas generation would leave more pores in the hardened matrix and reduce compactness, which explains the strength reduction observed when the hydrogen peroxide dosage was too high.

Therefore, the strength and roof-contact performance of the optimized backfill were controlled by a coupled mechanism of aggregate skeleton support, cementitious bonding and gas-generating expansion. The aggregate skeleton provided the basic load-bearing framework, hydration products enhanced particle bonding and pore filling, and hydrogen peroxide-induced gas expansion improved the active roof-contact ability. This synergistic mechanism allows the material to achieve both controlled expansion and sufficient mechanical strength, making it suitable for backfilling applications in end-slope mining.

## 4. Conclusions

In this study, an expansive solid-waste-based backfill material was prepared using mine overburden, ordinary Portland cement, fly ash and hydrogen peroxide for active roof contact in end-wall mining. The main conclusions are as follows:

(1) The prepared slurry showed good flowability and controllable expansion, with flowability ranging from 17.9 cm to 24.7 cm and expansion ratio ranging from 11.23% to 46.52%. The aggregate-to-binder ratio was the dominant factor affecting flowability, expansion ratio and 28 d compressive strength, while excessive hydrogen peroxide increased porosity and weakened compressive strength.

(2) The optimized mix proportion was determined as 40% fly ash dosage, an aggregate-to-binder ratio of 4:1 and 10% hydrogen peroxide dosage. This combination balanced slurry transportability, active roof-contact potential, long-term strength and solid-waste utilization.

(3) The optimized backfill exhibited clear age-dependent strengthening. With increasing curing age, the UCS and elastic modulus increased continuously, and the failure mode evolved from inclined shear failure to tensile splitting and Y-shaped tensile-shear composite failure.

(4) The shear behavior of the optimized backfill followed the Mohr-Coulomb criterion. The fitted shear strength equation was τ = 0.58σ + 1.61; the cohesion and internal friction angle were 1.61 MPa and 30.13°, respectively.

(5) SEM observations showed that the strength and expansion performance were governed by the synergistic mechanism of aggregate skeleton support, cementitious bonding and gas-generating expansion. This mechanism enables the material to achieve both active roof-contact capacity and mechanical support.

## Figures and Tables

**Table 1:** Mineral composition of the aggregate.

Mineral Composition	SiO_2_	CaAl_2_Si_2_O_8_	Al[Si_4_O_10_](OH)_8_	CaCO_3_
Content/%	62.8	30.9	3.9	2.5

**Table 2:** Mass fraction of aggregate particle size ranges for n = 0.5.

Grading Index (n)	Mass Fractions in Different Particle Size Intervals/%		
	0–1.0 mm	1.0–3.0 mm	3.0–5.0 mm
0.5	44.72	32.74	22.54

**Table 3:** Chemical composition contents of cement and fly ash.

Chemical Composition	SiO_2_	Al_2_O_3_	Fe_2_O_3_	CaO	MgO	Na_2_O	Other
Cement/%	24.49	7.45	4.07	55.82	1.97	1.03	5.17
Fly ash/%	43	24	2.5	5.7	0.93	/	22.97

**Table 4:** Factor levels selected from the preliminary single-factor experiments for the orthogonal design.

Test Level	Factor A	Factor B	Factor C	Error Column
	Fly Ash Dosage/%	Aggregate-to-Binder Ratio	Hydrogen Peroxide Dosage/%	
1	30	2	5	1
2	40	4	10	2
3	50	6	20	3

**Table 5:** Orthogonal experimental ratio design.

Group	Factor A	Factor B	Factor C	Error Column
	Fly Ash Dosage/%	Aggregate-to-Binder Ratio	Hydrogen Peroxide Dosage/%	
1	30	2	5	1
2	30	4	10	2
3	30	6	20	3
4	40	2	10	3
5	40	4	20	1
6	40	6	5	2
7	50	2	20	2
8	50	4	5	3
9	50	6	10	1

**Table 6:** Engineering-oriented multi-index selection strategy for the expansive backfill material.

Single-Index Target	Preferred Combination	Limitation for Engineering Application
Flowability	A2B1C2 (40% FA; 2:1; 10% H_2_O_2_)	Low aggregate-to-binder ratio increases binder consumption and reduces mine overburden utilization
Expansion ratio	A1B1C3 (30% FA; 2:1; 20% H_2_O_2_)	Excessive H_2_O_2_ dosage causes high porosity and strength loss
28 d compressive strength	A1B3C1 (30% FA; 6:1; 5% H_2_O_2_)	High aggregate-to-binder ratio reduces flowability and expansion capacity
Engineering-selected comprehensive performance	A2B2C2 (40% FA; 4:1; 10% H_2_O_2_)	Engineering trade-off among flowability, expansion, strength, solid-waste utilization, and binder economy; no weighted objective function was used

## Data Availability

The original contributions presented in this study are included in the article. Further inquiries can be directed to the corresponding author.
